# Prevalence of diabetic retinopathy in the United Arab Emirates: a cross-sectional survey

**DOI:** 10.1186/1471-2415-7-11

**Published:** 2007-06-16

**Authors:** Fatma Al-Maskari, Mohammed El-Sadig

**Affiliations:** 1 Department of Community Medicine, Faculty of Medicine & Health Sciences, United Arab Emirates University. Al-Ain, P.O. Box: 17666, U.A.E

## Abstract

**Background:**

Diabetic retinopathy (DR) is one of the leading causes of blindness. The aim of this study was to estimate the prevalence and determinants of retinopathy among diabetics in Al-Ain city, United Arab Emirates (UAE).

**Methods:**

The study was part of a general cross-sectional survey carried out to assess the prevalence of diabetes (DM) complications including retinopathy among known diabetic patients in Al-Ain District, UAE. Patients were randomly selected during 2003/2004. Patients completed an interviewer-administered questionnaire carried out by treating doctors and underwent a complete medical assessment. All patients were examined for evidence of diabetic retinopathy by ophthalmologist and their fundi were examined using slit lamp examination and fundus photography of each eye through dilated pupils.

**Results:**

A sample of 513 diabetic patients was selected with a mean age of 53 years (SD ± 13.01). Retinopathy was present in 19% (95% CI: 15.1–23.5%) of the study population. Most patients (74%) were not aware of their condition. The disease was more common among males (24.2 vs. 13.9%; p = 0.016), increased with increasing age (p = 0.004) and disease duration (p = 0.0001). Type I DM was a highly significantly contributing risk factor (38.3% for type 1, vs. 16.4%   for type 2; p < 0.0001). Retinopathy was higher among patients with hypertension, microalbuminuria, peripheral vascular disease, coronary artery disease and neuropathy.

**Conclusion:**

The prevalence of DR in the UAE was (19%) and significantly affected elderly males. Regular screening to detect DR is highly recommended as with the early detection of proliferative retinopathy and timely laser photocoagulation which are known to prevent most of the diabetes related blindness.

## Background

Diabetic retinopathy (DR) is a complication of diabetes mellitus (DM) that affects the blood vessels of the retina and leads to blindness. The progression of retinopathy is gradual, advancing from mild abnormalities, characterized by increased vascular permeability, to moderate and severe non-proliferative diabetic retinopathy, characterized by the growth of new blood vessels on the retina and posterior surface of the vitreous [1].

DR is one of the most serious complications of diabetes. For example, the Wisconsin epidemiological study of diabetic retinopathy (WESDR) concluded that 3.6% of those diagnosed with type 1, and 1.6% of those diagnosed with type 2 DM were legally considered blind. For type 1 DM, blindness was mostly (86%) due to diabetic retinopathy. For type 2 DM, blindness was related to retinopathy in 33% of the cases [2]. The prevalence of DR is probably around 30% in type 2 DM, but notably was above this level in five out of six studies reported from the Asian and pacific island nations of the Western Pacific Region [3]. The annual incidence of retinopathy requiring opthalmological follow up or treatment has been reported to average 1.5% after one year [4]. The same source estimates that 6–9% of patients with proliferative retinopathy or severe non proliferative disease would become blind each year [4]. Moreover, growing evidence also suggests that after 15 years of diabetes, approximately 2% of patients develop blindness, while about 10% develop severe visual handicap [4]. Thus, the early detection of sight-threatening retinopathy and the timely intervention with laser photocoagulation has been shown to be effective in preventing severe visual loss.

Several factors have been identified as determinants for the development of DR and its progression; including, type and duration of DM, age, gender, glycemic control, hypertension, body mass index (BMI), smoking, serum lipids and presence of microalbuminuria (MA) [[5-8] and [9]].

Despite the potential risk of DM complications in general and the grave burden imposed by DR in particular, no study was carried before to estimate the impact of the problem in the UAE. To our best knowledge this effort constitutes the first attempt to estimate the prevalence and determinants of DR among diabetics in Al-Ain district, UAE, with the specific objective of identifying high risk groups for DR amendable to timely intervention, in order to facilitate the planning of screening services. Al Ain city is located in the interior of Abu Dhabi Emirate and constitutes the second largest city (approximately 400,000 populations).

## Methods

### Overall design

The study was part of a general cross-sectional survey of DM patients carried out to assess and establish the prevalence of DM complications including retinopathy among diabetic patients in Al-Ain District, UAE during 2003/2004.

### Subjects and setting

Multi-stage random sampling was used to select 8 primary health care centers (PHC) in Al-Ain district (out of 22 rural and urban health care centers) in addition to two diabetic clinics in the only two governmental hospitals in the district (Tawam and Al-Ain hospitals). Thus, our sampling frame included all UAE national and non-national DM patients (types 1 and 2) of all ages of both genders, attending any of the selected primary health care centers (PHC) for any reason and diabetic clinics at the two hospitals for follow up. Within these primary sampling units (PHC and hospital clinics), a systematic random sampling was carried out to select a sub-sample of patients to be approached for participation in the study. In the absence of a computerized database in the UAE every third diabetic patient was approached to participate during the study period. A sample size of 625 was calculated to give a standard error in the prevalence of retinopathy of less than 2%. In total 600 patients were contacted by general practitioners and diabetologists, out of which, 513 patients (86%) agreed to enroll. The study was approved by the ethical committee of the Faculty of Medicine and Health Sciences of the UAE University.

### Data collection

After receiving informed consent, known diabetic patients in Al-Ain were interviewed by the treating doctors at PHC and hospital clinics and information pertinent to their DM type, duration, compliance with treatment as assessed by doctors, associated complications and co-morbidity was collected. Additionally, blood pressure was measured by the PHC/hospital nurse early in the morning and prior to drawing blood samples in the sitting position, using a standard mercury sphygmomanometer. Height was measured without shoes, and weight recorded while wearing indoor clothing. Body mass index (BMI) (weight in Kg, divided by height in meters squared) was calculated. The WHO (1977, 1979) classification for BMI was used to estimate the degree of obesity [10]. Fasting blood samples were taken to assess lipid profile, blood sugar and glycated hemoglobin (HbA_1C_) levels. Total lipid profile (total cholesterol (TC), high density lipoprotein (HDL), TC/HDL Ratio, low density lipoprotein (LDL) and triglycerides) were measured by a capillary tube whole blood method using the Cholesterol LDX lipid analyzer. Dyslipidaemia was taken to be present when the total cholesterol was > 5.60 mmol/L and/or triglycerides > 2.10 mmol/L, LDL > 3.4 mmol/L, and or HDL < 0.91 mmol/L [11]. Glycated hemoglobin (HbA_1C_) was measured using the Bayer DCA 2000+ analyzer and a value of less than 7% was taken to indicate good glycemic control. A standard 12- lead electrocardiogram (ECG) was recorded for all patients. The WHO definition of hypertension was used in this study: systolic blood pressure 160 mmHg or more and/or a diastolic blood pressure 95 mmHg or more, or on going treatment with antihypertensive drugs. MA was assessed using semi-quantitative dry immuno chemical screening strips (Micral 11 ^® ^test strips (Roche diagnostic GmbH Mannheim Germany). Micral Tests were performed on first morning urine sample collections and a value of more than 20 μg/min was judged as pathological.

All patients were referred to two ophthalmologists working at the two main hospitals of Al-Ain district and underwent detailed eye examination. After adequate mydriasis, the examination of the interior segment was carried out using Haag Streit slit lamp 900BQ with stereovariator. The intraocular pressure was measured using applanation tonometry. Fundus photography operating with a digital camera (Super 66 equipped with stereo fundus lens).

Diabetic retinopathy was classified using Watkins *et al *(2003) standards, and as follows: i) background retinopathy, if microaneurysms, haemorrhages (dot, blot or flamed shaped) or hard exudates and/or macular edema were present; ii) proliferative diabetic retinopathy, if cotton wool spots, multiple large blot haemorrhage, neovascularisation of the retina or iris, angle, venous beading, loops, and reduplication, arterial sheathing or atrophic looking retina were present; and iii) advanced diabetic eye disease, if vitreous haemorrhage, retinal detachment or rubeosis iriditis or glaucoma was present [12].

According to standard practice, dense cataract and blind patients with diabetes were also considered to have diabetic ophthalmopathy whenever it was impossible to establish the cause of their blindness. The severity of retinopathy was determined by the grading of the most seriously affected eye.

### Statistical analysis

The data was coded and processed on IBM compatible computers, using the Statistical Package for Social Sciences (SPSS) software (version 13). Descriptive analysis, using standard statistical methods was performed. Chi-square tests and Fisher exact tests were used to ascertain the association between retinopathy and clinical variables. The Mantel-Haenszel test was used to adjust relationships between categorical variables for dichotomous confounders, while logistic regression was used to estimate the simultaneous effect of several determinants on a dichotomous (yes/no) outcome.

## Results

### Socio-demographic characteristics of the study population

A total sample (n = 513) of diabetic patients of both gender and from all nationalities (UAE nationals and expatriates) resident in the Al-Ain district of Abu Dhabi emirate was selected. Of those, 52% were males, 27% were aged 60 years or above (mean age 53.3), 75% were UAE nationals and most patients (63%) were illiterate (Table 1). The data were collected between September 2003 and May 2004.

**Table 1:** Socio-Demographic   Characteristics of DM Patients in Al-Ain District, UAE, 2003-2004  (n=513)

**Variable name**	**Prevalence of DM**	

	**n**	**Percent (95% CI)**
**Sex**		
Male	264	51.5 (47.2–55.8)
Female	249	48.5 (44.2–52.8)

**Level of Education**		
Illiterate	318	62.8 (58.6–67.0)
Completed primary school	99	19.6 (16.1–23.1)
Completed secondary school	60	11.9 (9.1–14.7)
Completed university	29	5.7 (3.7–7.7)

**Age group (Years)**		
40 or less	81	15.8 (12.6–19.0)
41 – 49	137	26.8 (23–30.6)
50 – 59	154	30.1 (26.1–34.1)
60 or above	140	27.3 (23.4–31.2)

**Nationality Group**		
UAE	382	74.6 (70.8–78.4)
Other GCC citizens	54	10.5 (7.8–13.2)
Arabs from other countries	54	10.5 (7.8–13.2)
Asians	22	4.3 (2.5–6.1)

**BMI Group**		
Under weight	6	1.2 (0.2–2.2)
Healthy weight	113	22.5 (18.8–26.2)
Overweight and obese	383	76.3 (72.6–80.0)

### Clinical characteristics

Of the total sample, the majority (86%) had type 2 DM, 49% had been diagnosed incidentally and most of them (79%) had the disease for ≥ 10 years. Of the total sample 35% (95% C.I.: 30.8–39) had hypertension and 76% were obese or overweight.The analysis of glycemic control (HbA_1C_) showed that 38% (95%CI: 32.8–42.4) had good control (less than 7%), while it was poor in 62% (95%CI: 57.6–67.2). Dyslipidaemia, assessed by elevated total cholesterol, was present in 34.4% (95%CI: 30.0–38.8), and elevated triglycerides was present in 25.2% (95%CI: 21.1–29.3) (Table 2). Of the total sample, 12.8% (95%CI: 11.0–14.6) were current smokers while 8.2% (95%CI: 6.7–9.7) were ex-smokers.

**Table 2:** Clinical Characteristics of DM Patients in Al-Ain District, UAE, 2003-2004 (n=513)

**Variable name**	**Prevalence of DM**	
	**n**	**Percent (95% CI)**

**Type of DM**		
Type 1	68	13.6 (10.6–16.6)
Type 2	431	86.4 (83.4–89.4)

**Mode of Diagnosis**		
Incidental	245	48.5 (44.1–52.9)
Screening	39	7.7 (5.4–10.0)
Symptomatic	221	43.8 (39.5–48.1)

**Family History of DM**		
Present	270	54.3 (49.9–58.7)
Absent	227	45.7(41.3–50.1)

**Duration of the DM**		
< 1 year	33	6.6 (4.4–8.8)
1–5 years	199	40.0 (35.7–44.3)
6–10 years	161	32.3 (28.2–36.4)
11–20 years	98	19.7 (16.2–23.4)
> 21 years	7	1.4 (0.4–2.4)

**Hypertension**		
Present	178	34.9 (30.0–38.8)
Absent	332	65.1(61.0–69.2)

**Dyslipidaemia**		
High total cholesterol level	152	34.4 (30.0–38.8)
Within the reference range	290	65.5(61.1–69.9)

**Triglycerides**		
Elevated triglycerides	105	23.9 (19.9–27.9)
Within reference range	334	76.1(72.1–80.1)

**Microalbuminuria**		
Negative	175	38.8(34.3–43.3)
Positive	276	61.2(56.7–65.7)

**HbA**_1c_		
Good control	150	37.6(32.8–42.4)
Poor control	249	62.4(57.6–67.2)

### Prevalence of retinopathy

**Figure 1:**
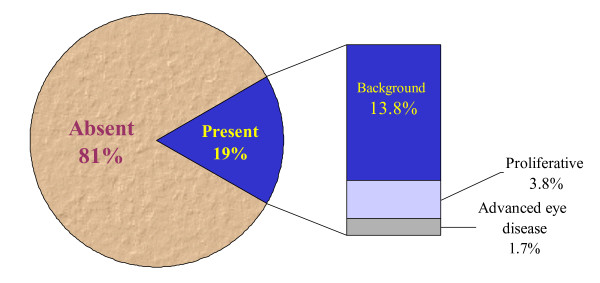
Retinopathy among   DM Patients in Al-Ain District, UAE, 2003-2004 (n=347).

Of the total study population, retinopathy was present in 19% (95% CI: 15.1–23.5). Only 1.5 % (5 cases) of the study sample were presumptive diagnosis due to the presence of cataract or blindness. Most patients detected with diabetic retinopathy (74%) were not aware of their eye condition before the survey. Of the total study sample, background retinopathy was present in 13.8%, proliferative retinopathy in 3.8% and advanced eye disease in only 1.7% (Figure 1). Diabetic retinopathy was significantly more common among males (24.7%) than females (13.9%) (p = 0.02). DR was significantly higher among type 1 DM patients (38.3%) compared to type 2 DM patients (16.4%) (p = < 0.001). The complication increased with increasing age (p < 0.01) and disease duration (p < 0.001) (Tables 3 and 4). Hypertension was marginally significantly associated with retinopathy (p = 0.07), but this was weakened somehow when adjusted for sex, age, and duration of disease (logistic regression adjusted OR 1.54, 95% CI 0.82–2.89). Microalbuminuria was significantly associated with diabetic retinopathy (p < 0.001), after adjustment for sex, age, and duration (logistic regression adjusted OR 2.04, 95% CI 1.03–4.07). The disease was statistically significantly associated with other chronic DM complications such as: i) peripheral neuropathy (p = 0.001), however, the association was only marginally statistically significant when adjusted for sex and duration of disease (logistic regression adjusted OR 1.70 95% CI 0.90–3.21); ii) coronary artery disease (p = 0.002), but again was only marginally statistically significant when adjusted for sex and duration of disease (adjusted OR 1.96 95% CI 0.91–4.22); and, iii) peripheral vascular disease (p = 0.009), however, this effect disappeared when adjusted for sex and duration of disease (Table 5). There was no significant association of DR with cerebrovascular disease, neither univariately, nor multivariately, but this may be due to the rarity of this condition in the sample population (3.5%). BMI, glycemic control, smoking and dyslipidaemia were not statistically associated with DR.

**Table 3:** Diabetic Retinopathy in   relation to Sociodemographic Characteristics of DM Patients in Al-Ain   District, UAE, 2003-2004 (n=513)

**Variable name**	**Presence of DR**		**Absence of DR**		
	**n**	**Percent (95% CI)**	**n**	**Percent (95% CI)**	**p-value**

**Sex**					0.016
Male	44	24.2(13.9–34.5)	138	75.8(70.8–80.8)	
Female	23	13.9(5.6–22.2)	142	86.1(82.0–90.2)	

**Level of Education**					0.147
Illiterate	43	20.6(10.8–30.4)	166	79.4(74.7–84.1)	
Completed primary school	8	12.1(4.2–20.0)	58	87.9(84.1–91.7)	
Completed secondary school	13	26.5(15.9–37.1)	36	73.5(68.3–78.7)	
Completed university	2	9.5(2.4–16.6)	2	90.5(87.1–93.9)	

**Age group (Years)**					0.004
40 or less	5	9.3(2.3–16.3)	49	90.7(87.3–94.1)	
41 – 49	15	15.0(6.4–23.6)	85	85.0(80.8–89.2)	
50 – 59	18	18.2(9.0–27.4)	81	81.8(77.3–86.3)	
60 or above	29	31.2(20.1–42.3)	64	68.8(63.4–74)	

**Nationality group**					0.005
UAE	49	19.7(10.2–29.2)	200	80.3(75.6–85.0)	
Other GCC citizens	2	5.9(0.3–11.5)	32	94.1(91.3–96.9)	
Arabs from other countries	7	15.9(7.1–24.7)	37	84.1(79.8–88.4)	
Asians	9	45.0(33.1–56.9)	11	55.0(49.2–60.8)	

**BMI Group**					0.166
Under weight	2	50.0(38.0–62.0)	2	50.0(44.1–55.9)	
Healthy weight	17	23.9(13.7–34.1)	54	76.1(71.1–81.1)	
Overweight and Obese	48	18.0(8.8–27.2)	218	82.0(77.5–86.5)	

**Table 4:** Diabetic Retinopathy in   relation to Clinical Characteristics of DM Patients in Al-Ain District, UAE,   2003-2004 (n=513)

**Variable name**	**Presence of DR**		**Absence of DR**		
	**n**	**Percent (95% CI)**	**n**	**Percent (95% CI)**	**p-value**

**Type of DM**					0.000
Type 1	18	38.3(26.6–50.0)	29	61.7(55.9–67.5)	
Type 2	48	16.4(7.5–25.3)	244	83.6(79.2–88.0)	

**Mode of Diagnosis**					0.596
Incidental	35	21(11.1–30.9)	132	79.0(74.2–83.8)	
Screening	3	13(4.8–21.2)	20	87.0(83.0–91.0)	
Symptomatic	27	18(8.7–27.3)	123	82.0(77.5–86.5)	

**Family History of DM**					0.282
Present	32	17.2(8.0–26.4)	154	82.8(78.3–87.3)	
Absent	33	21.9(11.8–32.0)	118	78.1(73.2–83.0)	

**Duration of the DM**					0.000
< 1 year	1	4.5(0–9.5)	21	95.5(93.0–98.0)	
1–5 years	9	6.7(0.6–12.8)	126	93.3(90.3–96.3)	
6–10 years	17	16.5(7.5–25.5)	86	83.5(79.1–87.9)	
11–20 years	36	52.2(40.1–64.3)	33	47.8(41.8–53.8)	
> 21 years	2	33.3(21.8–44.8)	4	66.7(61.1–72.3)	

**Hypertension**					0.050
Present	31	25.0(14.6–35.4)	93	75.0(69.9–80.1)	
Absent	36	16.3(7.5–25.1)	185	83.7(79.4–88.0)	

**Dyslipidaemia**					0.936
High total cholesterol level	22	20.0(10.2–29.8)	88	80.0(75.1–84.9)	
Within the reference range	42	19.6(9.9–29.3)	172	(75.6–85.2)	

**Triglycerides**					0.500
Elevated triglycerides	14	17.1(7.8–26.4)	68	82.9(78.3–87.5)	
Within reference range	49	20.5(10.5–30.5)	190	79.5(74.6–84.4)	

**Microalbuminuria**					
Negative	27	23.5(12.3–34.7)	88	76.5(71.2–81.8)	0.073
Positive	28	15.2(5.7–24.7)	156	84.8(80.3–89.3)	

**HbA**_1C_					0.556
Good control	21	36.2(23.8–48.6)	97	40.4(34.2–46.6)	
Poor control	37	63.8(51.4–76.2)	143	59.6(53.4–65.8)	

**Table 5:** Diabetic Retinopathy in   relation to Systemic Diseases among DM Patients in Al-Ain District, UAE,   2003-2004 (n=513)

**Associated Systemic Diseases**		**Diabetic Retinopathy**			
		**Present (n = 67)**	**Absent (n = 280)**		
	
**Type**	**N (%)**	**n**	**n**	**Adjusted OR**	**95% CI**

Microalbuminuria	276(61.2%)	28	156	2.04	(1.03–4.07)
Hypertension	178(34.9%)	31	93	1.54	(0.82–2.89)
Peripheral Neuropathy	199(38.8%)	35	80	1.70	(0.90–3.21)
Coronary Artery Disease	73(14.2%)	18	33	1.96	(0.91–4.22)
Peripheral Vascular Disease	59(11.5%)	16	32	1.19	(0.52–2.75)

In a multivariate stepwise logistic regression (with backward selection of variables) of retinopathy on age, sex, duration of diabetes and hypertension, only the female gender (adjusted OR 0.573, 95% CI 0.297–1.107), age (adjusted OR 1.029 per year; 95% CI 01.002–1.056), type of DM (adjusted OR for type 2 0.468, 96%CI 0.198–1.103) and, duration of DM (adjusted OR 1.013 per year, 95% CI 1.008–1.018) were found to be (marginally) significantly associated with retinopathy.

## Discussion

The International Diabetes Federation (IDF) in 2003 ranked the UAE's prevalence rates for type 2 DM and impaired glucose tolerance (IGT) as the second highest in the world (20% for DM and 26% for IGT) [3] implying that the disease and its complications such as retinopathy might constitute a sizable health care burden to the UAE population. Despite that, little is known about the true impact of DM and its complications, including diabetic retinopathy, in the UAE population. While the prevalence of DR in Al-Ain, UAE (19%) is clearly lower than that reported in other populations such as the US (40–45 % [13]), Saudi Arabia (31%), Oman (42%) and Egypt (42%) [14,15] and [16], it was substantially higher than the equivalent rate reported in a similar study in Kuwait (8%) [17]. The substantial heterogeneity in reported prevalence of retinopathy may partly be real, for example due to differences in the age structure of different population, but may also be due to differences in study methodology and population sample.

Our findings clearly demonstrate that, as elsewhere, DR is a common health problem and may well be among the leading causes of blindness in UAE adults. It is well established that nearly all patients with DM – of both type 1 and type 2 – are at high and increasing risk for the disease [2,13]. The fact that the majority of our study population (63%) were illiterate further reveal and emphasize the seriousness and complexity of the problem in the UAE. Therefore, additional care needs to be taken to illiterate patients to have a comprehensive annual eye examination with pupil dilation while treating doctors should also be reminded to closely follow DM treatment guidelines and refer all diabetics to ophthalmologist for management and treatment as indicated to preserve vision.

The analysis of the sample population showed that DR increases with patients' age and disease duration, which is consistent with findings elsewhere [5,6,11,12], and [18]. Comparatively, the duration of diabetes is known to reflect total glycemic control and risk factors exposure over time [2,18]. While this may suggest avenues for primary prevention, the true prospects for that are currently unknown. For example, Aiello *et al *[13] in a longitudinal study have shown that after 20 years duration, nearly all type 1 DM patients and approximately two thirds of type 2 DM end up developing retinopathy regardless of their diabetic control. However, the analysis revealed that DR in the study sample was marginally significantly associated with hypertension, which is largely held as a significant risk factor in most studies [7,19]. Thus, improved monitoring and control of hypertension among DM patients in the UAE, which has always been shown to slow the progression of retinopathy, particularly among those with poorly controlled diabetes, is strongly recommended [20].

As reported elsewhere, the presence of microalbuminuria was highly significantly associated with DR in the study population (OR 2.04; 95% CI: 1.03–4.07). MA is a well known predictor for diabetes cardiovascular complications and therefore, DM patients should be targeted for screening for early detection and treatment [21,22].

The prevalence of retinopathy was also higher in DM patients with other chronic DM complications such as coronary artery disease (p = 0.002), peripheral neuropathy (p = 0.001) and peripheral vascular disease (p = 0.009). However, a multivariate stepwise logistic regression analysis on these chronic complications showed only marginal significant association after adjusting for sex and duration which is consistent with studies in the neighboring countries such as Sultanate of Oman [23]. Surprisingly, and unlike findings elsewhere [18], the degree of glycemic control did not show any significant association with DR in the study sample.

Our study has few limitations. First, the assumption that DM patients with blindness and cataract had diabetic retinopathy as the primary cause, may have slightly led to some over estimation of the prevalence of retinopathy. Second, it is known that DM is notoriously under-diagnosed, and therefore while our sample probably adequately reflects DR among diagnosed DM patients; it is likely that this proportion still reflects only the top of the iceberg.

## Conclusion

Our study documented for the first time the prevalence, type and determinants of diabetic retinopathy in the UAE. This complication was related to the patient's age, male gender, type of DM, disease duration and presence of hypertension, MA and other chronic complications of diabetes. As such it is important to recommend introducing early screening program for DR for all diabetic patients, and especially those with associated hypertension, microalbuminuria and those with long disease duration.

Notwithstanding that the rate of DM in the population is high, and the fact that a high rate of undiagnosed diabetes exits in the UAE, it is highly recommended that urgent efforts should be made to improve health education to DM patients. It is established that the knowledge of the retinopathy status of an individual is just one in a whole process of care in diabetes. The implicit 'gold standard' for identifying and grading retinopathy remains, the retinal examination by the ophthalmologist. Several studies [2,24] have demonstrated the cost effectiveness of screening for retinopathy and established that screening for DR saves vision at a relatively low cost; much lower and safe than with later interventions like involving intra-ocular surgery in the absence of a screening program. Equally, the enormous potential debilitating indirect costs of retinopathy on patients could be curbed with increased productivity and decreased disability. Much awareness, management and attention to the complication is therefore needed in the UAE.

## Competing interests

The author(s) declare that they have no competing interests.

## Authors' contributions

F Al-Maskari conceived the need for the survey in the UAE, participated in its design, and contributed to the interpretation of the results. M El-Sadig participated in the design of the study and the data analysis. F Al-Maskari and M. El-Sadig collaborated in writing up the manuscript. All authors read and approved the final manuscript.

## Pre-publication history

The pre-publication history for this paper can be accessed here:


